# The neuroprotective *γ*-hydroxybutyrate analog 3-hydroxycyclopent-1-enecarboxylic acid does not directly affect CaMKII*α* autophosphorylation at T286 or binding to GluN2B

**DOI:** 10.1016/j.molpha.2025.100029

**Published:** 2025-03-12

**Authors:** Carolyn Nicole Brown, Rachel E. Blaine, Chase Madison Barker, Steven J. Coultrap, Karl Ulrich Bayer

**Affiliations:** Department of Pharmacology, University of Colorado Anschutz Medical Campus, Aurora, Colorado

**Keywords:** CaMKII, Cerebral ischemia, GluN2B, LTP, Synapse

## Abstract

The Ca^2+^/calmodulin (CaM)-dependent protein kinase II (CaMKII) mediates physiological long-term potentiation (LTP) of synaptic strength and pathological ischemic neuronal cell death. Both functions require CaMKII autophosphorylation at T286 (pT286) and binding to the NMDA-type glutamate receptor subunit GluN2B. The neuroprotection seen with 3-hydroxycyclopent-1-enecarboxylic acid (HOCPCA) was thought to be mediated by impairing binding of the brain-specific CaMKII*α* isozyme to GluN2B. However, we show that HOCPCA does not inhibit CaMKII*α* enzymatic activity, pT286, cocondensation with GluN2B, or binding to GluN2B. Consistent with no effect on GluN2B binding in vitro or in HEK293 cells, HOCPCA also did not affect the CaMKII*α* movement to excitatory synapses in hippocampal neurons in response to LTP stimuli. These findings leave the neuroprotective mechanism of HOCPCA unclear but explain why HOCPCA does not impair LTP.

**Significance Statement:**

This study found that the neuroprotective compound 3-hydroxycyclopent-1-enecarboxylic acid (HOCPCA) does not directly interfere with Ca^2+^/calmodulin (CaM)-dependent protein kinase II (CaMKII) activity or GluN2B binding. Although this leaves the neuroprotective mechanism of HOCPCA unclear, it explains why HOCPCA does not impair long-term potentiation. Overall, this limits the use of HOCPCA as a tool compound to study CaMKII functions, but not its clinical potential.

## 1 Introduction

The Ca^2+^/calmodulin (CaM)-dependent protein kinase II (CaMKII) forms 12 meric holoenzymes and is best known for its physiological functions in synaptic plasticity, specifically including in long-term potentiation (LTP; Bayer and Schulman, 2019; Yasuda et al, 2022; Bayer and Giese, 2025). However, CaMKII also mediates pathological ischemic neuronal cell death (Vest et al, 2010; Coultrap et al, 2011; Deng et al, 2017). LTP induction requires both CaMKII autophosphorylation at T286 (pT286) and binding to GluN2B (Giese et al, 1998; Tullis et al, 2023; Rumian et al, 2024). The expression phase of LTP additionally requires CaMKII-mediated phosphorylation of exogenous substrates activity (Rumian et al, 2024). Notably, both pT286 and GluN2B directly generate Ca^2+^-independent “autonomous” activity that outlasts the initial Ca^2+^/CaM-stimulus (Miller and Kennedy, 1986; Bayer et al, 2001). Similar to LTP, ischemic neuronal cell death can also be prevented by pharmacological inhibitors of CaMKII, by knockout of the CaMKII *α* isozyme, by CaMKII T286A mutation that prevents pT286, or by the GluN2B^ΔCaMKII^ mutation that prevents CaMKII binding to GluN2B (Vest et al, 2010; Deng et al, 2017; Buonarati et al, 2020). Interestingly, in mouse models of stroke, neuroprotection was also seen with a CaMKII*α*-binding compound that does not appear to inhibit enzymatic CaMKII activity: the *γ*-hydroxybutyrate analog, 3-hydroxycyclopent-1-enecarboxylic acid (HOCPCA; Vogensen et al, 2013; Leurs et al, 2021; Griem-Krey et al, 2023). Although HOCPCA was found to stabilize the CaMKII holoenzyme at extreme temperatures, its effects on physiological and pathophysiological CaMKII mechanisms that confer neuroprotection remain unclear. Initial studies suggested that HOCPCA may interfere with CaMKII binding to GluN2B after excitotoxic glutamate in dissociated neuron cultures (Leurs et al, 2021), which should indeed confer neuroprotection (Buonarati et al, 2020). However, CaMKII binding to GluN2B binding is also required for LTP, and LTP is not impaired by HOCPCA (Leurs et al, 2021). To shed light on this apparent conundrum, we decided to test the direct effects of HOCPCA on CaMKII activity, pT286, and GluN2B binding. We found no effect of HOCPCA on CaMKII activity or pT286 in vitro, as expected. However, our results also indicated that HOCPCA does not block GluN2B binding and allows for normal CaMKII movement to excitatory synapses in hippocampal neurons in response to LTP stimuli. These findings leave the neuroprotective mechanism of HOCPCA unclear but explain why HOCPCA does not impair LTP.

## 2 Materials and methods

### 2.1 Experimental models and subject details

All animal treatments were approved by the University of Colorado Anschutz Medical Campus Institutional Animal Care and Use Committee, in accordance with National Institutes of Health guidelines. Animals are housed at the University of Colorado Anschutz Medical Campus Animal Resource Center and are regularly monitored for general health, cage changes, and overcrowding. Pregnant Sprague-Dawley rats were obtained from Charles River Labs. Dissociated hippocampal neuron cultures were prepared on postnatal day 0 (P0) and imaged on days in vitro (DIV) 15–18.

### 2.2 Material and DNA constructs

Material was obtained from Sigma Aldrich, unless noted otherwise. HOCPCA was obtained from Drs. Petrine Wellendorph and Bente Frølund; the synthesis has been described previously (Vogensen et al, 2013). Expression of all constructs is driven by the CMV promoter with the exception of the intrabody, which is driven by the CAG promoter. The constructs for the intrabody (Gross et al, 2013; Cook et al, 2019), CaMKII expression (Bayer et al, 2006), GluN2B expression (Goodell et al, 2017), and CaMKII shRNA knockdown (Barcomb et al, 2014) have been described previously. Note that all CaMKII constructs are fusions with mEGFP, that is, EGFP with additional A206K mutation to further minimize GFP dimerization, which is especially important for multimeric proteins such as CaMKII. Otherwise, they contain the same linker region between the GFP and CaMKII as the originally described construct (Shen and Meyer, 1999), specifically coding for the amino acid sequence SGLRSRAQASNSAVDGTAGPGS.

### 2.3 Protein purification

Expression and purification of CaMKII*α*, CaM, and glutathione *S*-transferase (GST)-GluN2B was conducted according to established protocol described in detail previously (Bayer et al, 2001; Cook et al, 2021). CaMKII*α* was purified from a baculovirus/Sf9 cell expression system. CaM and GST-GluN2B were purified after expression in BL21 bacteria.

### 2.4 Assay of CaMKII activity and pT286

Phosphate incorporation into peptide substrates was assessed as described (Coultrap et al, 2010). Assays were done at 30 °C for 1 minute and contained CaMKII holoenzymes at 2.5 nM concentration of kinase subunits, 50 mM 1,4-piperazinediethanesulfonic acid (PIPES), pH 7.1, 0.1% bovine serum albumin (BSA), 10 mM MgCl_2_, 100 *μ*M [*γ*-^32^P]ATP (∼1 mCi/mmole), and 75 *μ*M syntide-2 substrate peptide. Stimulated activity was measured for naïve CaMKII in the presence of CaCl_2_ (1 mM) and CaM (1 *μ*M); autonomous activity assays contained EGTA (1.5 mM) instead. CaMKII (100 nM) T286 prephosphorylation was done in stimulation buffer, but without substrate and ^32^P, for >5 minutes on ice. Before activity assays, pT286 autophosphorylated CaMKII activity was halted, and CaM was dissociated by diluting the kinase 8-fold and adding 5 mM EDTA for at least 5 minutes on ice. Kinase reactions were stopped by spotting onto P81 cation exchange chromatography paper (Whatman) squares and immersing in 0.5% phosphoric acid. After extensive washes in water, phosphorylation of the substrate peptide bound to the P81 paper was measured by liquid scintillation counting.

To determine the effects of HOCPCA on pT286, CaMKII*α* (100 nM) was autophosphorylated as above, except that reactions were carried out for 15 seconds at 30 °C in the presence or absence of HOCPCA (2 mM). We used the shorter 15-second reactions for autophosphorylation, as the longer 1-minute reactions are typically near maximal for the autophosphorylation reactions that have the same “kinase” and “substrate” concentration (although they are linear for reactions with excess of external substrate), which has the potential to miss minor drug effects due to a ceiling effect of the reaction. The reactions were stopped by diluting them into a sample buffer containing 5 mM EDTA, and phosphorylation of pT286 was determined by western blot.

### 2.5 Western blots

Western blots were performed as described previously (Cook et al, 2021; Tullis et al, 2023). Before undergoing SDS-PAGE, the samples were heated in a sample buffer (final concentrations of 54 mM Tris pH 6.8, 1.6% SDS, 8% glycerol, 50 mM dithiothreitol, and 0.013% bromophenol blue) for 5 minutes at 95 °C. Proteins were separated using gradient precast gels (BioRad) and then transferred to low-fluorescence polyvinylidene difluoride membranes for 1 hour at 4 °C. Membranes were blocked in a fluorescent blocking buffer (Azure) and then incubated with anti-CaMKII (1:1000, BD Biosciences) or anti-CaMKII*α* (1:2000, CB*α*2, made in house), anti-GST (1:1000, Millipore), and anti-pT286 CaMKII (1:3000, Phosphosolutions). After incubation with fluorescent secondary antibodies, antimouse AzureSpectra 700 (1:10,000, Azure Biosciences) or antirabbit AzureSpectra 800 (1:10,000, Azure Biosciences) signal was measured by fluorescent imaging using an Azure Western Blot Imaging System. Densitometric quantification was performed using Fiji ImageJ.

### 2.6 In vitro GluN2B binding assay

CaMKII binding to the GluN2B C-tail was assessed similarly as previously described (Bayer et al, 2001; Rumian et al, 2024). Anti-GST coated 96-well plates were washed 3 times in wash buffer that is here termed PST for its content of (in mM) 50 PIPES pH 7.12, 150 NaCl, and 0.1% Tween-20. GST-GluN2Bc (diluted in PST containing 0.05% BSA) was added in saturating amounts to wells for 1 hour under gentle agitation at room temperature and washed 3 times with PST. Wells were blocked in 5% BSA in PST for 30 minutes under gentle agitation at room temperature. CaMKII (50 nM subunits; either purified nontagged holoenzymes or mEGFP-CaMKII from HEK293 cell lysates) was bound for 30 minutes in kinase binding buffer containing (final concentration, in mM) 50 PIPES pH 7.12, 150 NaCl, 1.0 MgCl_2_, 0.1 ADP, 0.05% BSA, 0.05% Tween-20, and Ca^2+^/CaM (2 mM/1 *μ*M). The supernatant was discarded, and the wells were washed 4 times in PST containing EGTA (1 mM). Wells were then incubated in a gel loading buffer containing 1.6% SDS at 95 °C for 10 minutes to dissociate the bound proteins.

### 2.7 Transfection of neurons and HEK293 cells

DIV 14–16 rat neuronal cultures were transfected with Lipofectamine 2000 (Invitrogen) to express mCherry-PSD-95 intrabody and mEGFP-CaMKII, using 0.5 *μ*g of DNA per well in a 12-well plate, unless specifically indicated otherwise. HEK293 cells were transfected with 1.5 *μ*g of each plasmid per well in a 12-well plate using the calcium phosphate method for 24 hours before imaging experiments.

### 2.8 Assay of CaMKII binding to GluN2B in HEK293 cells

Live imaging of HEK293 and image analysis were performed at 34 °C in a HEPES-buffered imaging solution containing: 130 mM NaCl, 5 mM KCl, 10 mM HEPES (pH 7.4), 20 mM glucose, 2 mM CaCl_2_, and 1 mM MgCl_2_. Ionomycin was added at a final concentration of 10 *μ*M after baseline images were collected and left in the imaging chamber for the duration of the experiment. Postionomycin images were taken every minute for 5 minutes following ionomycin stimulation. For analysis, a cell filled with a nuclear export signal was used to generate a mask of the cytoplasm. Colocalization of mEGFP-CaMKII and pDisplay-mCherry-GluN2B (WT and S1303A) within the mask was quantified using Pearson’s correlation.

### 2.9 Assay of CaMKII cocondensation with GluN2B in HEK293 cells

HEK293 cells were transfected and imaged as described above. However, in this case, mEGFP-CaMKII was coexpressed with a soluble, nonmembrane-targeted version of the mScarlet-GluN2B-c tail. Cocondensate formation was induced by triggering a Ca^2+^ stimulus with 10 *μ*M ionomycin for 6 minutes; dispersal or maintenance of the cocondensates was monitored after subsequent chelation of Ca^2+^ with 2.5 mM EGTA for an additional 5 minutes at 34 °C in an imaging buffer. HEK293 cells were preincubated for 1 hour with 2 *μ*M HOCPCA where indicated. For analysis, the CaMKII intensity ratio, defined as the fluorescent intensity within CaMKII puncta divided by the total fluorescent intensity within the cell per time point, was used to quantify the dynamics of cocondensate formation and maintenance. As the level of coclustering was negatively correlated with the level of CaMKII expression, we eliminated low and high-expressing cells from the analysis in order to ensure that the 2 conditions were compared in cells with similar medium levels of CaMKII expression.

### 2.10 Primary hippocampal culture preparation

Hippocampi were dissected from mixed sex rat pups on P0, dissociated in papain solution for 1 hour, and plated at 100,000 cells/mL on 18 mm #1 coverslips in plating media (MEM containing 10% FBS, 1% Penicillin-Streptomycin) in 12-well plates. On DIV 1, the plating media was replaced with a feeding media (Neurobasal A containing 2% B27 and 1% Glutamax). On DIV 7, half of the conditioned feeding media was replaced with a fresh feeding media containing 2% 5-Fluoro-2’-deoxyuridine to control the growth of glial cells.

### 2.11 Live imaging of neurons and image analysis

Rat neuronal cultures were imaged 24–48 hours after transfection on DIV 15–16. Chemical LTP (cLTP) was induced by bath application of 100 *μ*M glutamate, and 10 *μ*M glycine in artificial cerebrospinal fluid (ACSF) for 30 seconds, followed by 4× volume washout with fresh ACSF. Cells were imaged using a 63× objective on a Marianas fast spinning disk confocal imaging system by Intelligent Imaging Innovations (3i) controlled by SlideBook software. During image acquisition, neurons were maintained at 34 °C in ACSF solution containing (in mM): 130 NaCl, 5 KCl, 10 HEPES pH 7.4, 20 glucose, 2 CaCl_2_, and 1 MgCl_2_, adjusted to proper osmolarity with sucrose. Pre and every minute for 5 minutes post-cLTP, washout images were collected. Two dimensional maximum intensity projection images were then generated, and the mean mEGFP intensity (CaMKII) at excitatory (PSD-95) synapses was quantified, as well as the mean intensity of the dendritic shaft. Synaptic CaMKII is defined by the ratio of synaptic (PSD-95)/shaft CaMKII. Total changes in CaMKII synaptic accumulation were determined by subtracting the post–pre-synaptic CaMKII. All representative images were prepared using Fiji software (ImageJ, National Institutes of Health).

### 2.12 Quantification and statistical analysis

All data are shown as mean ± SEM. Statistical significance is indicated in each figure legend. Statistical analyses were performed using Prism (GraphPad) software. Imaging experiments were obtained and analyzed using SlideBook 6.0 and Fiji ImageJ software. All comparisons between 2 groups with independent samples were analyzed using unpaired, two-tailed Student’s *t* tests. Comparisons between 3 or more groups were done by one-way ANOVA with specific post-hoc analysis indicated in figure legends. Comparisons between 3 or more groups with 2 independent variables were assessed by 2-way ANOVA with Bonferroni post-hoc test to determine whether there is an interaction and/or main effect between the variables. Asterisks represent level of significance: ∗*P* < .05, ∗∗*P* < .01, ∗∗∗*P* < .001, ∗∗∗∗*P* < .0001; ns, not significant.

## 3 Results

### 3.1 HOCPCA has no effect on stimulated or autonomous CaMKII activity

**Fig. 1:**
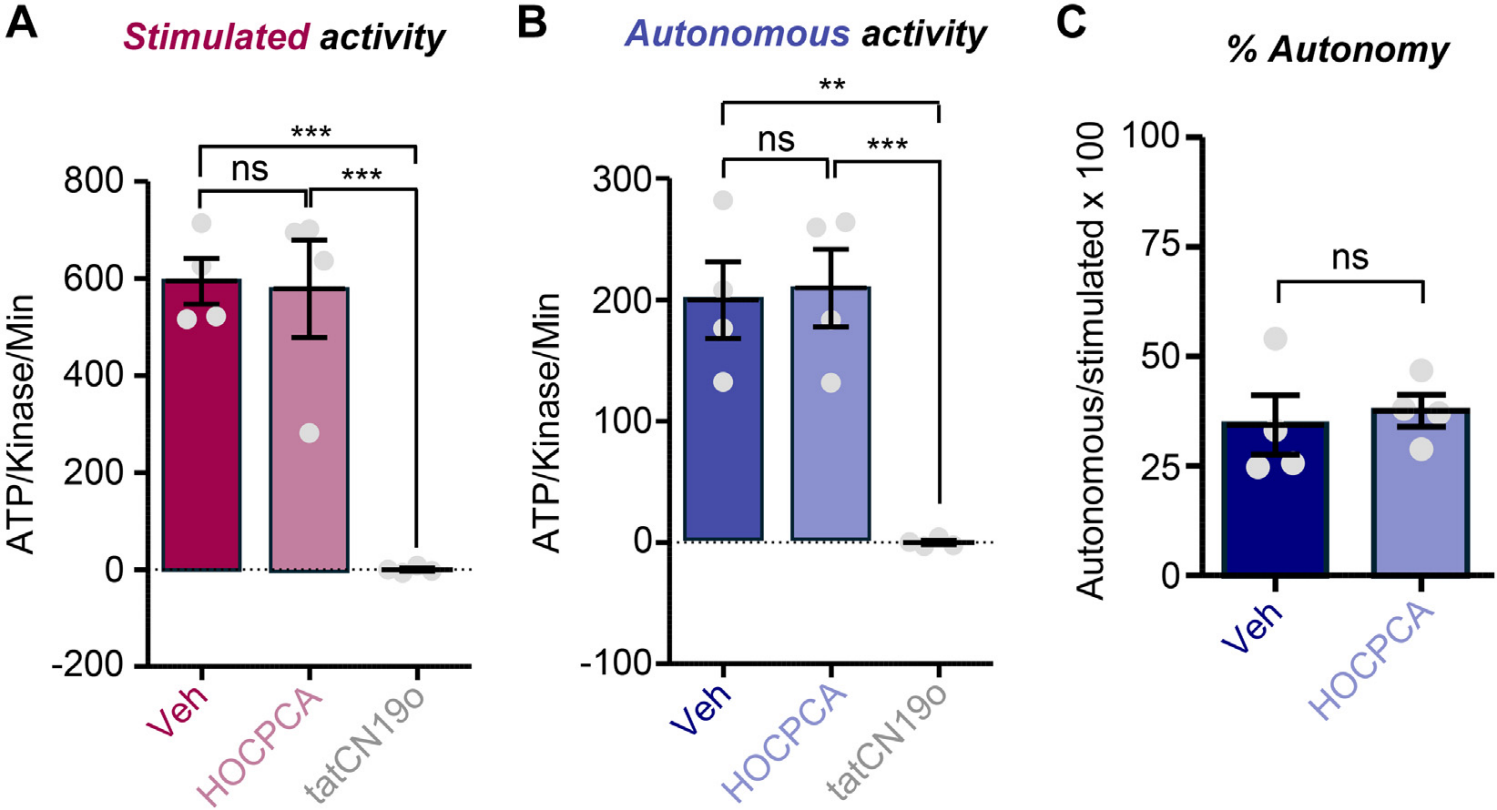
No effect of HOCPCA on CaMKII stimulated or autonomous activity. Kinase reactions were performed with purified CaMKII*α* (2.5 nM) with or without HOCPCA (2 mM) or tatCN19o (200 nM). Shown is quantification of CaMKII activity after 1-minute reaction times (in nM ATP/nM kinase subunits/min) with vehicle, HOCPCA, or tatCN19o. (A) CaMKII activity was stimulated with Ca^2+^/CaM (1 mM/1 *μ*M) and measured by ^32^P-phosphate incorporation into the syntide-2 (75 *μ*M) substrate. ∗∗∗*P* < .001; ns, not significant by 1-way ANOVA. Direct comparison of vehicle vs HOCPCA by *t* test did not yield significance either. (B) pT286-dependent autonomous CaMKII activity was measured by ^32^P-phosphate incorporation into the syntide-2 (75 *μ*M) substrate. ∗∗*P* < .01; ∗∗∗*P* < .001; ns, not significant by 1-way ANOVA. Direct comparison of vehicle vs HOCPCA by *t* test did not yield significance either. (C) Autonomous activity, that is, the ratio of autonomous activity over stimulated activity indicated in percent. ns, not significant by unpaired *t* test.

Previous studies indicated that HOCPCA does not affect enzymatic activity of CaMKII but relied on prolonged reaction times (>45 minutes) and an indirect method that assesses ATP depletion (Leurs et al, 2021; Narayanan et al, 2024). Thus, we decided to test the effect of HOCPCA in our method that uses purified CaMKII*α* in 1-minute reactions and directly measures the incorporation of ^32^P-labeled phosphate into the substrate peptide syntide 2 under linear reaction conditions (Coultrap et al, 2010). Additionally, we compared the effect on stimulated versus autonomous CaMKII activity, with stimulation directly induced by Ca^2+^/CaM and autonomous activity measured after pT286 and subsequent chelation of Ca^2+^. As expected from previous studies, autonomous activity was substantially lower than stimulated activity (Fig. 1), although with ∼35% of stimulated activity, the level of autonomous activity was somewhat on the higher end, at least for regular CaMKII substrates such as syntide 2 (Coultrap et al, 2010, 2014; Woolfrey et al, 2018). Neither form of activity was affected by HOCPCA even at a concentration of 2 mM, whereas the CaMKII inhibitor peptide tatCN19o completely blocked both forms of activity at 0.2 *μ*M (Fig. 1, A and B). Consequently, HOCPCA also did not change the level of relative autonomy, that is, the ratio of autonomous overstimulated activity (Fig. 1C). These results confirm previous conclusions that even high concentrations of HOCPCA do not inhibit Ca^2+^/CaM-stimulated activity of CaMKII*α*; additionally, they demonstrate that HOCPCA does also not inhibit the autonomous activity of CaMKII*α* that is generated by pT286.

### 3.2 HOCPCA has no effect on pT286

**Fig. 2:**
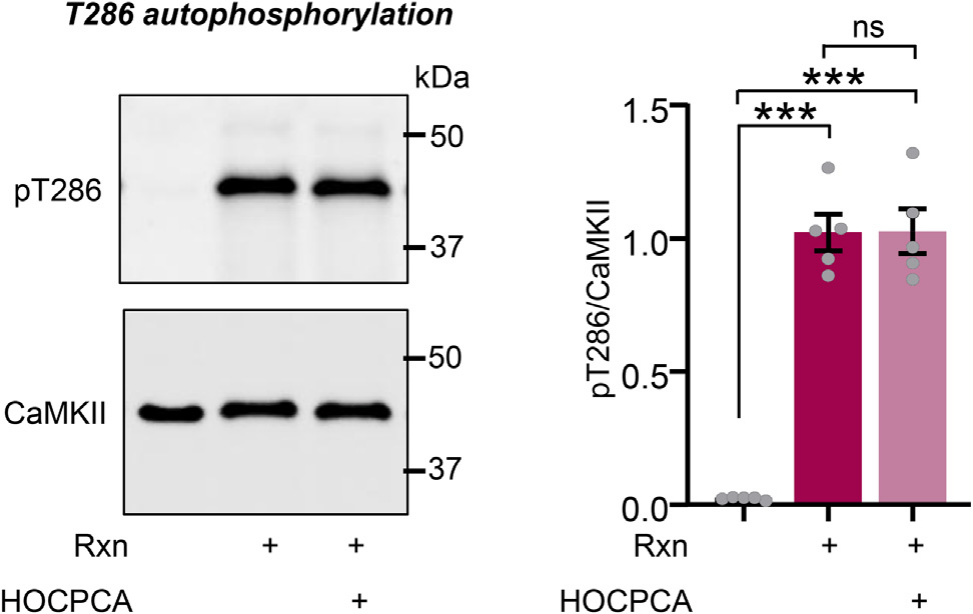
No effect of HOCPCA on pT286. Kinase reactions were performed with purified CaMKII*α* (100 nM) with or without HOCPCA (2 mM). CaMKII and pT286 were detected by western blot. Shown are representative blots and quantification of pT286/CaMKII after 15 seconds reaction times (Rxn) with vehicle or HOCPCA. Each data point is the average of 1 reaction sample analyzed by 2 Western blots. ∗∗∗*P* < .001; ns, not significant by 1-way ANOVA. Direct comparison of vehicle vs HOCPCA by *t* test did not yield significance either.

CaMKII pT286 occurs between the subunits within the 12-meric CaMKII holoenzyme (Hanson et al, 1994; Rich and Schulman, 1998). Thus, as HOCPCA binding could have effects on the holoenzyme, it could potentially alter pT286 even if it does not affect the enzymatic activity of an individual CaMKII subunit. Thus, we decided to determine the effect of HOCPCA on CaMKII pT286 in in vitro kinase reactions with purified CaMKII*α* in the presence of 2 mM HOCPCA or vehicle. Western blot of the resulting pT286 indicated no effect of HOCPCA on pT286 (Fig. 2). Thus, HOCPCA inhibits neither the pT286-generated autonomous CaMKII activity nor the pT286 reaction itself.

### 3.3 HOCPCA has no effect on CaMKIIα binding to GluN2B in vitro

**Fig. 3:**
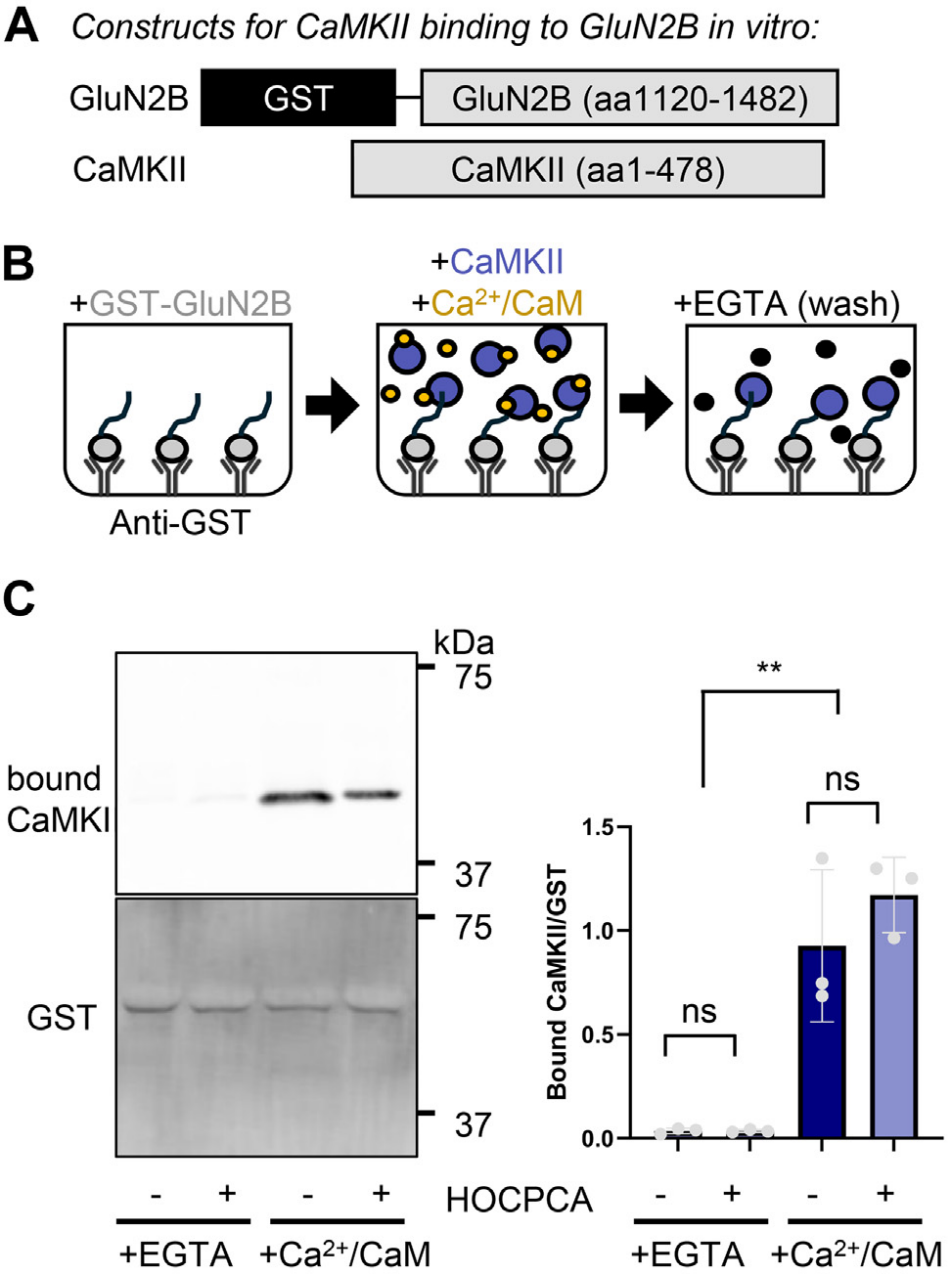
No effect of HOCPCA on CaMKII binding to GluN2B in vitro. (A) Constructs used in this experiment. (B) Schematic of experiment. GST-GluN2B is bound to an anti-GST coated plate. CaMKII was added to the plate in the presence of Ca^2+^/CaM (or EGTA) and unbound kinase was then washed away with EGTA. Bound samples were analyzed by western blot. (The apparent negative stain above the GST signal is likely the BSA protein that is used as blocking agent in the binding assay.) (C) Representative blot and quantification of bound CaMKII corrected for total bound GST-GluN2B. CaMKII binding was induced by Ca^2+^/CaM and was unaffected by HOCPCA (by 1-way ANOVA with Tukey post-hoc analysis; ns, not significant, ∗∗*P* < .001; direct comparison of the conditions with and without HOCPCA by *t* test also yielded no significant difference).

In order to determine the potential effect of HOCPCA on CaMKII*α* binding to GluN2B, we first used our established in vitro CaMKII-GluN2B binding assay. Purified CaMKII*α* was added to anti-GST coated 96-well plate wells containing immobilized GST-GluN2B C-tail (GST-2BC; aa1122-1482; Fig. 3, A and B). In order to induce binding, Ca^2+^/CaM (2 mM/1 *μ*M) and nucleotide (100 *μ*M ADP) were also present (Bayer et al, 2001; O'Leary et al, 2011). The amount of bound CaMKII was determined by western blot (Fig. 3C). As expected, CaMKII binding was induced by Ca^2+^/CaM and not seen in the presence of EGTA. Most importantly, no difference in the amount of bound CaMKII was detected in the presence of 2 mM HOCPCA compared with control (Fig. 3C). HOCPCA also showed no effect on Ca^2+^/CaM-induced CaMKII binding when binding was tested using mEGFP-CaMKII*α* (Supplemental Fig. 1). Thus, HOCPCA does not affect CaMKII*α* binding to GluN2B in our biochemical in vitro binding assay.

### 3.4 HOCPCA has no effect on CaMKIIα binding to GluN2B in HEK293 cells

**Fig. 4:**
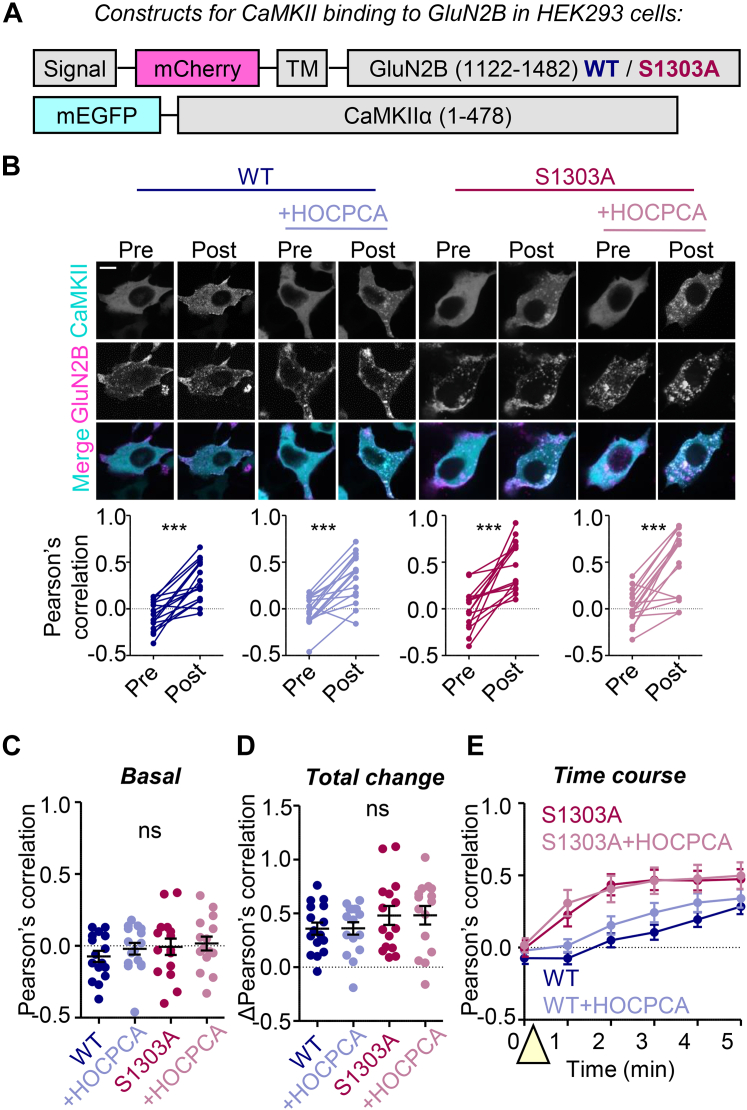
No effect of HOCPCA on CaMKII binding to GluN2B in HEK293 cells. (A) Constructs used in this experiment. Here, a membrane-targeted version of GluN2B is used. (B) Representative images and paired quantification of vehicle- or HOCPCA-treated CaMKII/GluN2B WT or S1303A colocalization before (pre) and after (post) ionomycin stimulation. ∗∗∗*P* < .001 by paired *t* test. (C) Basal colocalization of CaMKII and GluN2B with and without HOCPCA treatment; ns, not significant by 2-way ANOVA with Bonferroni post-hoc analysis. (D) Total change (post–pre) in colocalization of CaMKII and GluN2B with and without HOCPCA treatment. ns, not significant by 2-way ANOVA with Bonferroni post-hoc analysis. (E) Time course of vehicle- or HOCPCA-treated CaMKII/GluN2B colocalization upon ionomycin stimulation (indicated with yellow triangle.

Next, we tested CaMKII*α* binding to GluN2B within HEK293 cells using our established colocalization assay of overexpressed mEGFP-CaMKII*α* with membrane-targeted mCherry-GluN2B C-tail (Goodell et al, 2017; Buonarati et al, 2020; Tullis et al, 2023; Rumian et al, 2024; Fig. 4, A and B). CaMKII and GluN2B colocalization was measured by Pearson’s correlation before and after induction of a Ca^2+^-stimulus by the addition of 10 *μ*M ionomycin (Fig. 4B). Preincubation with 2 mM HOCPCA for 1 hour affected neither the basal colocalization of CaMKII with GluN2B before ionomycin treatment (Fig. 4C) nor the induction of colocalization by ionomycin (Fig. 4, D and E). In addition to GluN2B wild-type, we tested the GluN2B S1303A mutant, as the binding of CaMKII to this mutant lacks the negative regulation by S1303 phosphorylation that can be mediated by enzymatic CaMKII activity (Strack et al, 2000; O’Leary et al, 2011). Again, HOCPCA had no effect on CaMKII colocalization with this GluN2B mutant in HEK293 cells (Fig. 4, B–E). Although the binding of CaMKII was faster for the GluN2B S1303A mutant compared with wild-type, HOCPCA did not affect this time course for either condition (Fig. 4E). Thus, HOCPCA does not inhibit CaMKII*α* binding to GluN2B, neither in our biochemical nor in our cellular assay.

### 3.5 HOCPCA has no significant effect on CaMKIIα cocondensation with GluN2B in HEK293 cells

**Fig. 5:**
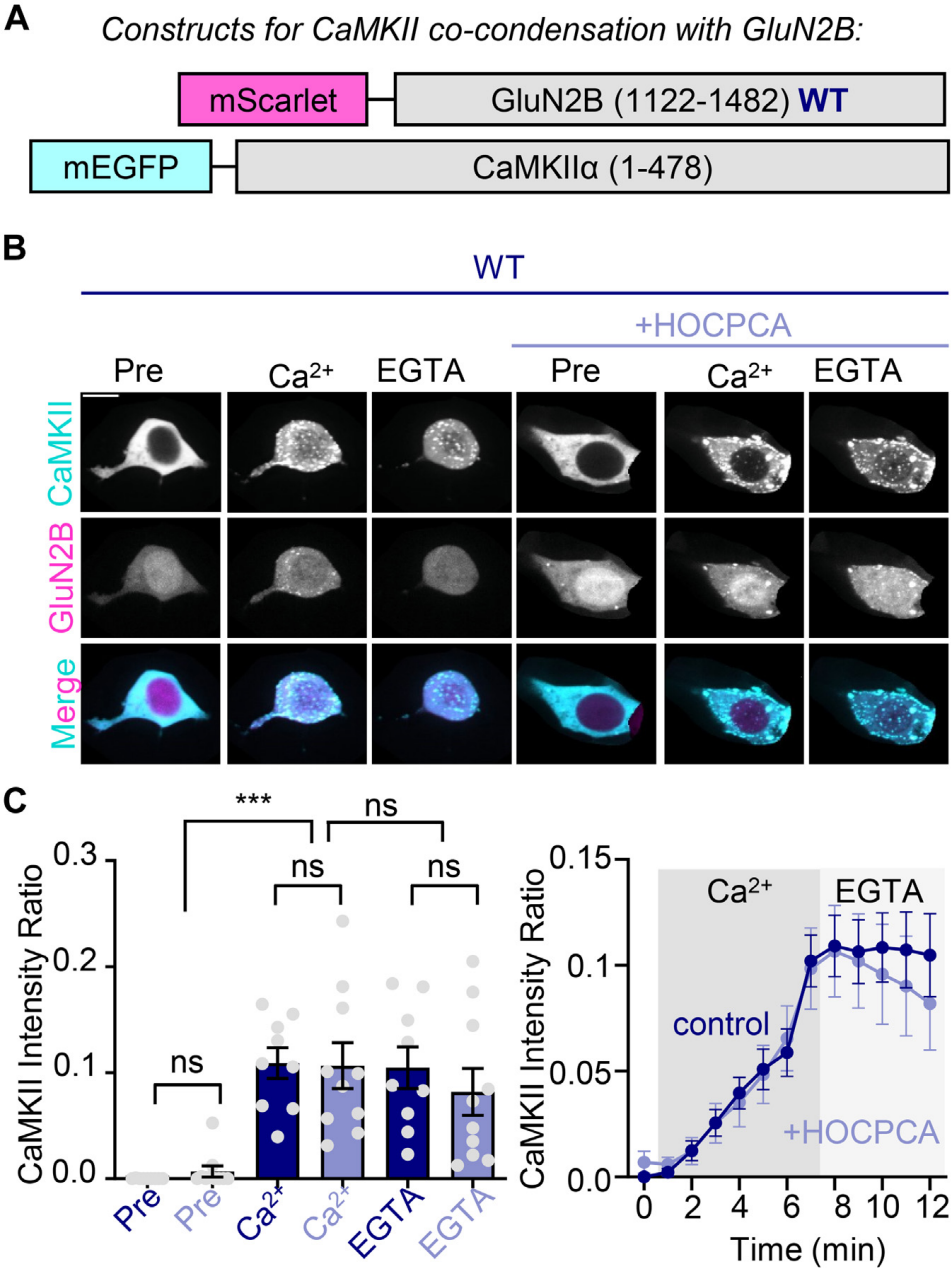
No significant effect of HOCPCA on cocondensation of CaMKII with GluN2B in HEK293 cells. (A) Constructs used in this experiment. Here, a soluble version of GluN2B is used. (B) Representative images of vehicle- or HOCPCA-treated CaMKII/GluN2B WT cocondensation before (pre) and after (+Ca^2+^) ionomycin stimulation, as well as after calcium chelation with EGTA (EGTA). (C) Quantification of cocondensation of CaMKII and GluN2B with and without HOCPCA treatment at the example time points (left) and in the overall time course of the experiment (right). Cocondensation was induced by Ca^2+^ and then maintained after chelation of Ca^2+^ with EGTA; although there appeared to be slightly less Ca^2+^-induced cocondensation after the HOCPCA treatment, the difference was not statistically significant (by 2-way ANOVA with Bonferroni post-hoc analysis; ns, not significant, ∗∗∗*P* < .001; direct comparison of the conditions with and without HOCPCA by *t* test also yielded no significant difference).

In addition to the traditional binding interaction, CaMKII*α* and GluN2B can also form cocondensates (Hosokawa et al, 2021; Cai et al, 2021), which are typically thought to be mediated by multiple low-affinity interactions (Hyman et al, 2014; Banani et al, 2017; Alberti et al, 2019; Chen et al, 2020). Thus, we tested HOCPCA also for its effects on the cocondensation of CaMKII with GluN2B in our recently developed HEK293 cell assay, which is similar to the binding assay but instead utilizes the expression of a GluN2B C-tail that is not membrane-anchored but soluble in the cytoplasm (Rumian et al, 2024; Fig. 5A). Consequently, both CaMKII and GluN2B are dispersed throughout the cell before stimulation but then forms clusters upon initiating a Ca^2+^-stimulus by the addition of 10 *μ*M ionomycin, an effect that remained the same also when the cells were preincubated with 2 mM HOCPCA for 1 hour (Fig. 5, B and C). Although the extent of cocondensation appeared slightly diminished by HOCPCA in the time course after chelation of Ca^2+^ with EGTA (Fig. 5C), this effect was not statistically significant, neither by the appropriate analysis by 2-way ANOVA (see Fig. 5B) nor when the time points were directly compared by *t* test (not shown). Thus, HOCPCA blocks neither CaMKII binding nor cocondensation with GluN2B.

### 3.6 HOCPCA has no effect on CaMKII accumulation at excitatory synapses after LTP stimuli

**Fig. 6:**
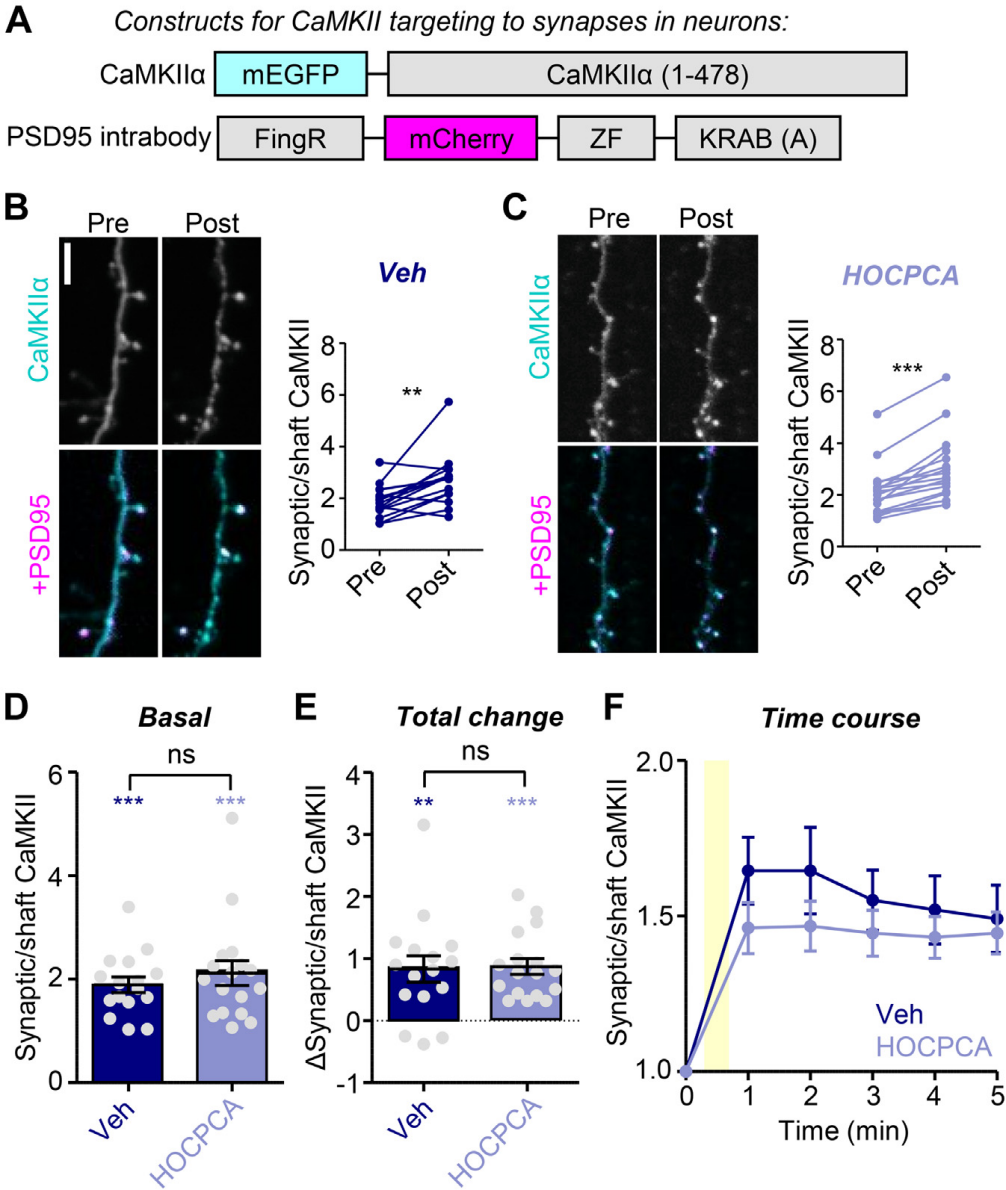
No effect of HOCPCA on CaMKII synaptic accumulation during chemical LTP. (A) Constructs used in this experiment. (B) Representative images and paired quantification of vehicle- or HOCPCA-treated neurons before and 5 minutes after bath application of chemical LTP (cLTP; 100 *μ*M glutamate, 10 *μ*M glycine, 30 seconds). ∗∗*P* < .01, ∗∗∗*P* < .001, paired *t* test. (C) Basal synaptic CaMKII in Vehicle vs HOCPCA-treated cells. Colored asterisks: ∗∗∗*P* < .001 with 1 sample *t* test vs hypothetical mean = 1. ns, not significant by unpaired *t* test. (D) Total change (post–pre) in synaptic CaMKII upon cLTP application. Colored asterisks: ∗∗*P* < .01, ∗∗∗*P* < .001 with 1 sample *t* test vs hypothetical mean = 1. ns, not significant by unpaired *t* test. (E) Normalized time course of synaptic accumulation of CaMKII. Yellow area indicates time of cLTP application before washout.

Finally, we measured the movement of mEGFP-labeled CaMKII*α* to excitatory synapses in response to LTP stimuli in dissociated hippocampal neuron cultures, a movement that requires the regulated binding of CaMKII to GluN2B (Bayer et al, 2001; Barria and Malinow, 2005; Halt et al, 2012). In parallel to expressing mEGFP-labeled CaMKII*α*, endogenous CaMKII*α* was knocked down using an established shRNA (Barcomb et al, 2014). Excitatory synapses were identified using intrabodies against PSD95, and synaptic CaMKII enrichment was determined by the ratio CaMKII colocalized with the PSD95 label over CaMKII localized in the dendritic shaft. The constructs used are illustrated in Figure 6A. As expected, synaptic CaMKII enrichment increased significantly after chemical LTP stimuli (cLTP; 100 *μ*M glutamate, 10 *μ*M glycine, 30 seconds; Fig. 6B). Significant CaMKII movement was also detected when 2 mM HOCPCA was added 1 hour prior to the cLTP stimulus (Fig. 6C). Direct comparison between the conditions with and without HOCPCA indicated that HOCPCA had no effect on CaMKII localization, neither on the basal localization before stimulation nor on the change in localization at 5 minutes after stimulation (Fig. 6D). Any possible effect on the overall time course of CaMKII movement was minor and not statistically significant (Fig. 6E). Thus, overall, we found no indication for an effect of HOCPCA on CaMKII binding to GluN2B in vitro, in HEK293 cells, or in neurons.

## 4 Discussion

This study showed that the neuroprotective *γ*-hydroxybutyrate analog HOCPCA does not directly inhibit enzymatic CaMKII activity, CaMKII autophosphorylation at pT286, CaMKII binding to GluN2B, CaMKII cocondensation with GluN2B, or CaMKII movement to excitatory synapses in response to chemical LTP stimuli. These results explain why HOCPCA does not inhibit hippocampal LTP, but they leave the mechanism by which HOCPCA may mediate neuroprotection unclear. Our study was motivated by prior findings that showed specific binding of HOCPCA to the brain-specific CaMKII*α* isoform and suggested that HOCPCA may interfere with the colocalization of CaMKII and GluN2B after excitotoxic stimuli (Leurs et al, 2021), which should indeed result in neuroprotection (Buonarati et al, 2020). Based on these findings, we expected that HOCPCA may disrupt CaMKII binding to GluN2B without affecting enzymatic activity. Such properties would substantially extend the pharmacological toolbox available to study CaMKII, as current pharmacological compounds either inhibit both enzymatic activity and GluN2B binding or only enzymatic activity (Brown and Bayer, 2024). Thus, an inhibitor of GluN2B binding that does not affect enzymatic activity would provide a valuable complement. Notably, based on recent findings, such a compound would still be expected to block LTP (Tullis et al, 2023; Rumian et al, 2024), an effect that is not caused by HOCPCA (Leurs et al, 2021), thereby further validating our conclusions.

At first glance, our findings of normal CaMKII movement to synapses in hippocampal neurons in the presence of HOCPCA appear to be in conflict with the previous report of reduced CaMKII cocolocalization with GluN2B, especially since both studies used slightly different but overall very similar glutamate stimuli (Leurs et al, 2021). However, closer examination reveals that the 2 studies measured fundamentally different parameters: although our study assessed the increase in CaMKII that is colocalized with PSD95 in dendritic spines, the previous study instead assessed the colocalization with GluN2B in dendritic shafts (Leurs et al, 2021), an analysis that favors extrasynaptic GluN2B. Notably, although CaMKII movement to excitatory synapses requires direct binding to GluN2B, an increase in colocalization with extrasynaptic GluN2B may be mediated by other interactions. Indeed, it has been reported that glutamate stimuli can lead to a transient association of CaMKII with microtubule that may be mediated by binding to MAP2 (Lemieux et al, 2012), the same protein that was used as a dendritic marker in the previous study (Leurs et al, 2021). In either case, any potential effect of HOCPCA on CaMKII association with GluN2B in neurons would have to be indirect, as our studies in both test tubes and HEK293 cells rule out a direct effect of HOCPCA on CaMKII binding to GluN2B.

Unfortunately, until a clear mechanism of action is established, HOCPCA will not be a useful tool for studying CaMKII. However, this does not negate its therapeutic potential in protecting neurons from ischemic cell death (Leurs et al, 2021; Griem-Krey et al, 2023), a property that HOCPCA shares with compounds that block enzymatic CaMKII activity (Vest et al, 2010; Coultrap et al, 2011; Deng et al, 2017). In fact, HOCPCA has 2 potential advantages over inhibitors of enzymatic CaMKII activity: its isoform-selectivity and its lack of an effect on LTP. However, isoform selectivity is more relevant for chronically used inhibitors, and not so much for acute use in conditions such as cerebral ischemia. Moreover, for chronic use, drug developers typically try to specifically target isoforms other than the brain-specific *α* isoform. This is due to the expectation that chronic inhibition of CaMKII*α* in the brain would have detrimental effects on learning, memory, and cognition, due to the central role of CaMKII in LTP (Bayer and Schulman, 2019; Yasuda et al, 2022; Bayer and Giese, 2025). However, recent studies indicate that inhibition of CaMKII with tatCN19o (which also inhibits the CaMKII*α* isoform) has only a mild and very transient detrimental effect on learning, with no effect on memory at all (Rumian et al, 2023). Thus, chronic treatment may be possible after all even with inhibitors of CaMKII*α*.

However, what could be the mechanisms of action of HOCPCA? Binding to CaMKII*α* remains the most likely target, supported by radioligand binding studies in brain slices from wild-type mice compared with CaMKII*α* knockout (Leurs et al, 2021). HOCPCA selectively binds the CaMKII*α* isozyme with high nanomolar to low micromolar affinity (Leurs et al, 2021). Based on structural consideration, the binding site is expected to be on the CaMKII association domain that holds the holoenzyme together (Leurs et al, 2021), which is also consistent with the lack of an effect on the enzymatic activity that is mediated by the kinase domains. Thus, it has been proposed that HOCPCA stabilizes the CaMKII holoenzyme. Indeed, HOCPCA protects CaMKII protein from unfolding at high temperatures (Leurs et al, 2021). However, it is unclear what effect HOCPCA would have on holoenzyme stability at physiological temperatures, especially considering the remarkable stability of CaMKII holoenzymes even at very low, subphysiological concentrations (Torres-Ocampo et al, 2020). One possibility is that HOCPCA treatment prevents the proposed CaMKII subunit exchange by stabilizing the holoenzyme in its normal dodecameric structure (Stratton et al, 2014). However, recent experiments have challenged the idea of such subunit exchange (Lučić et al, 2023), making it unclear whether this process occurs and what role it may play in physiology or pathophysiology. One proposed physiological role of subunit exchange has been to mediate LTP maintenance by maintaining the pT286 state of CaMKII. However, if HOCPCA prevents CaMKII subunit exchange, then the lack of an effect by HOCPCA on LTP provides evidence against the role of such subunit exchange in LTP. Indeed, there is now mounting evidence that pT286 plays a role in LTP induction, but not in maintenance (Tullis et al, 2023; Rumian et al, 2024; Bayer and Giese, 2025).

Perhaps consistent with the unclear mechanism and rather mild effect on CaMKII, the neuroprotective effects of HOCPCA have been somewhat mixed. For example, HOCPCA showed different levels of protection against excitotoxicity-induced cell death in cultured neurons from different mouse strains (Leurs et al, 2021). Additionally, the effect of HOCPCA on infarct volume differed after various different stroke models (Leurs et al, 2021; Griem-Krey et al, 2023); however, HOCPCA consistently showed improvement in behavioral outcome measures (Leurs et al, 2021; Griem-Krey et al, 2023). Thus, the therapeutic effects of HOCPCA remain promising. However, until the underlying CaMKII-directed mechanisms are clarified, the utility of HOCPCA as a tool compound remains limited. Fortunately, with the current 3 mechanistically distinct classes of CaMKII inhibitors, the pharmacological toolbox to study CaMKII functions is now richer than ever before (Brown and Bayer, 2024).

## Conflict of interest

K.U.B. is cofounder and board member of Neurexis Therapeutics, a company that seeks to develop a CaMKII inhibitor into a therapeutic drug for cerebral ischemia. C.N.B., S.J.C., and K.U.B. are named inventors on patent applications related to cerebral ischemia that were submitted by the Regents of the University of Colorado.
