# Supplementary material for: The neuroprotective γ-hydroxybutyrate analog 3-hydroxycyclopent-1-enecarboxylic acid does not directly affect CaMKIIα autophosphorylation at T286 or binding to GluN2B
Source: Mol Pharmacol. 2025 Mar 12;107(4):100029. doi: 10.1016/j.molpha.2025.100029 (PMC13095473; doi:10.1016/j.molpha.2025.100029)
Supplement: Supplementary Figure 1 [file mmc1.pdf]

Article title:
The neuroprotective γ-hydroxybutyrate analog HOCPCA does not directly affect CaMKIIα autophosphorylation at T286 or binding to GluN2B

Authors:
Carolyn Nicole Brown, Rachel E. Blaine, C. Madison Barker, Steven J. Coultrap, and K. Ulrich Bayer[‡]

Journal title:
Molecular Pharmacology

Manuscript number:
MOLPHA-D-24-00008

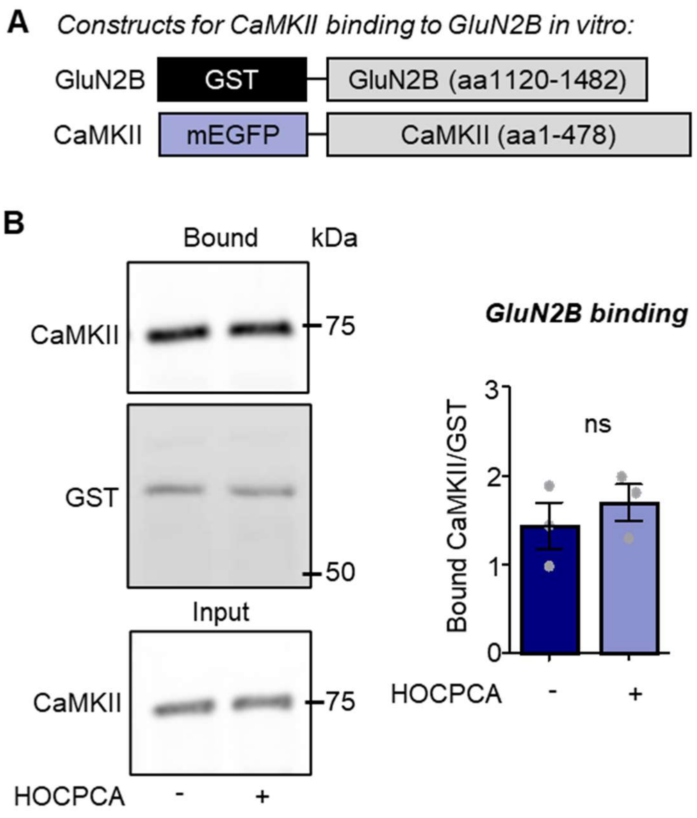

Supplemental Fig 1. No effect of HOCPCA on mEGFP-CaMKII binding to GluN2B *in vitro*. Similar to Fig. 3, but testing binding of mEGFP-CaMKII expressed in HEK293T cells instead of non-tagged, purified CaMKII.

A) Constructs used in this experiment.

B) Representative blot and quantification of bound mEGFP-CaMKII corrected for total bound GST-GluN2B. ns, not significant by unpaired t-test.
